# A Measurement Method for Large Parts Combining with Feature Compression Extraction and Directed Edge-Point Criterion

**DOI:** 10.3390/s17010040

**Published:** 2016-12-26

**Authors:** Wei Liu, Yang Zhang, Fan Yang, Peng Gao, Zhiguang Lan, Zhenyuan Jia, Hang Gao

**Affiliations:** Key Laboratory for Precision and Non-Traditional Machining Technology of the Ministry of Education, Dalian University of Technology, No. 2 LingGong Road, Dalian 116024, China; yangyangcici@mail.dlut.edu.cn (Y.Z.); yangfandut@mail.dlut.edu.cn (F.Y.); gaopeng3412@mail.dlut.edu.cn (P.G.); lanzhg1009@mail.dlut.edu.cn (Z.L.); Jzyxy@dlut.edu.cn (Z.J.); gaohang@dlut.edu.cn (H.G.)

**Keywords:** binocular vision system, feature extraction, active triangulation scanning measurement

## Abstract

High-accuracy surface measurement of large aviation parts is a significant guarantee of aircraft assembly with high quality. The result of boundary measurement is a significant parameter for aviation-part measurement. This paper proposes a measurement method for accurately measuring the surface and boundary of aviation part with feature compression extraction and directed edge-point criterion. To improve the measurement accuracy of both the surface and boundary of large parts, extraction method of global boundary and feature analysis of local stripe are combined. The center feature of laser stripe is obtained with high accuracy and less calculation using a sub-pixel centroid extraction method based on compress processing. This method consists of a compressing process of images and judgment criterion of laser stripe centers. An edge-point extraction method based on directed arc-length criterion is proposed to obtain accurate boundary. Finally, a high-precision reconstruction of aerospace part is achieved. Experiments are performed both in a laboratory and an industrial field. The physical measurements validate that the mean distance deviation of the proposed method is 0.47 mm. The results of the field experimentation show the validity of the proposed method.

## 1. Introduction

High-accuracy three-dimensional (3D) measurement for the surfaces of large objects plays a crucial role in evaluating the safe performance of large aviation parts. The geometrical shape of measured parts directly affects assembly quality. High-accuracy 3D measurement becomes more challenging because of the significant increases of the sizes of major equipment parts. Taking composite skins of aircraft horizontal and vertical tails as examples, they need to be precisely measured to ensure the quality of assembly. Due to the fact that large aviation composite parts are mostly weakly rigid, the parts should be placed on an assembly tool to ensure the part shape. Thus, on-site measurement is required. Moreover, there is requirement for high surface quality of the aviation part, thus the surface of the part should not be pasted anything to prevent the deterioration of the surface quality. In addition, because the scale of aviation parts is always large, the parts should be measured by using a convenient measuring method within finite time in complex on-site environments. Therefore, research in high-accuracy and high-speed on-site measurement for such surfaces is significant for the manufacturing of large aviation parts.

Visual measurement has gradually become an active research area owing to its inherent advantages of non-contact, high accuracy and high speed. Thus, visual measurement is widely used in the measurement of aviation parts [1,2]. At present, three main visual measurement methods are utilized to measure aviation parts: photogrammetry with reflective marks [3,4,5]; profilometry with projected grating; and machine vision with laser stripes [6,7,8,9]. Photogrammetry with reflective marks is a measurement method of high accuracy and stability. However, to measure large parts using this method, numerous markers need to be affixed to the measured surfaces, which is labor-intensive and time-consuming. Furthermore, the sticky material on the marks is difficult to completely remove, which results in damage of the measured surfaces. Profilometry with projected grating uses fewer images than other methods, however its calculation is relatively complicated. In addition, the illumination intensity of the projected grating is low and the field depth is small, so the grating image cannot be obtained in environments under complex illumination. Machine vision with laser stripes is a scanning measurement method, in which the surfaces of parts are reconstructed through scanning laser stripes. With the lasers’ advantages of high brightness, parallelism, and stability, images with high-quality laser stripes can be obtained under complex illumination. Furthermore, relative motion between the objects and cameras is not required during using the active binocular measurement method, so this method is suitable for measuring large aviation parts.

Since the centers of the scanning laser stripes are commonly defined as the feature information to reconstruct the surface, the laser stripe center extraction has become a research hotspot. At present, two kinds of extraction methods are used to achieve the stripe extraction: the fitting extraction method based on the Gaussian characteristics of laser stripe and the direct extraction method based on the geometrical characteristic of laser stripe. The fitting extraction method is proposed by Steger [10]. He presented an approach to extract curvilinear lines by using an asymmetric parabolic or Gaussian line model to fit the line profiles. Normally, the high-precision measurement results cannot be obtained without large amount of calculation. Lukas proposed that initial positions of stripe centers could be extracted roughly, and then the centers could be extracted precisely through Gauss fitting of the neighbor 5 pixels in the cross section directions. It had the characteristics of high extraction accuracy for linear stripes as well as a low extraction speed caused by Gauss fitting in each column (row) of the stripes [11]. For improving the measurement accuracy, Steger proposed the Hessian matrix method to get the normal directions of stripes in images. Thus, the extraction of sub-pixel centers is achieved. Then, several corrections and improvements were proposed to make the method more suitable for the computation of normal directions. Bazen [12] proposed the method based on principle component analysis (PCA) to estimate high resolution directional field of an image. Cai [13] utilized the PCA algorithm to obtain the normal directions of the laser stripe. Then the centers could be found in normal directions with second order Taylor series expansion of grayscale distribution function. Liu [14] detected the normal directions of the centerline based on the characteristics of the first derivative of the light stripe and then located the center points in sub-pixel level along the normal directions by using the pixel subdivision and the centroid of gray method. However, these extraction methods cost much time of computation, and the laser stripe in the image should keep the high quality of Gaussian characteristics. However, in the process of measurement for large aircraft parts, the brightness distribution of the laser stripe is not uniform. Moreover, due to the influences of complex environments, the image quality is relatively poor. Thus, the direct extraction methods based on the geometrical characteristic of laser stripe are mostly adopted by current commercial measurement equipment in the field of aircraft assembly, such as the centroid of gray method (COG), geometric center method and maximum value method. The most common method is the method of centroid of gray (COG) [15], which is proposed by Haug [16] and Usamentiaga [17]. This method could get a better extraction results and simultaneously meet the requirements of efficiency and robustness. However, this method is only sub-pixel in one dimension. The measurement accuracy of this method is poor, especially in the boundary of part. The boundary of part is represented by the edge points of laser stripes. Since the edge points of laser stripe have more noise than other part of laser strip, they have lower extraction accuracy than other points. With improving requirement of accuracy, the methods mentioned above cannot guarantee the stability, speed and accuracy of extraction for laser stripes in the sequence images. Moreover, for better analysis of aviation parts, the measurement results should contain more information, including the boundary information of the parts. However, the current commercial measurement equipment cannot be efficiently used in the measurement process of aviation composite part assembly. For solving the above problems, a non-contact measurement method, with high measurement accuracy and speed and containing boundary information, is proposed in this paper.

This paper is organized as follows: Section 2 expounds on the measurement principle for large aviation parts. Section 3 describes in detail the extraction and reconstruction algorithms used in the proposed system. Section 4 discusses the laboratory and field experiments, which are conducted to verify the effectiveness of the proposed system. Section 5 concludes the method proposed in this paper.

## 2. Measurement Principle of the Method

The 3D measuring system for large aviation parts utilizes active triangulation scanning measurement based on the binocular stereovision principle. The system consists of two high-speed and high-resolution CMOS cameras, a high-stability line-structured laser projector, and a high-performance image work station. In the process of measurement, a scanning laser stripe is projected on the surface of measured part. Sequential images of the laser stripe are captured by the two cameras. Then, the conversion relation between two-dimensional (2D) image coordinate and 3D space coordinate is established based on the camera calibration. Centers of the laser stripes extracted from the captured images can be reconstructed in the world coordinate system according to calibration results. Therefore, the surface of the measured object is reconstructed based on reconstructions of the scanning laser stripes.

**Figure 1:**
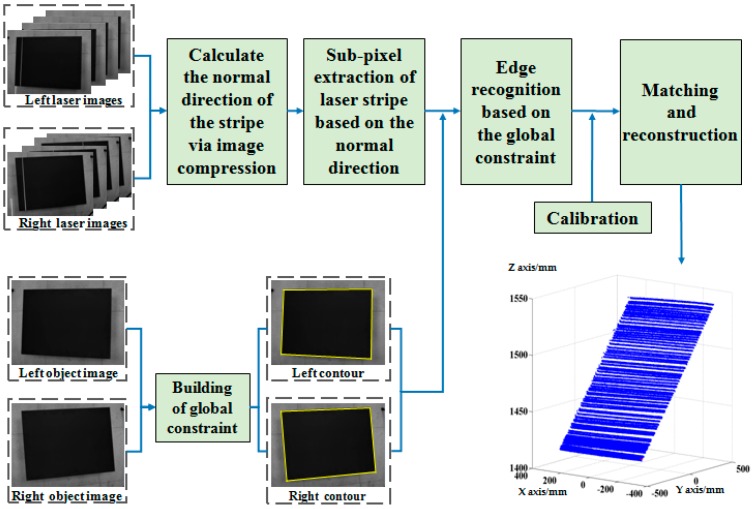
Block diagram of the proposed measuring method.

**Figure 2:**
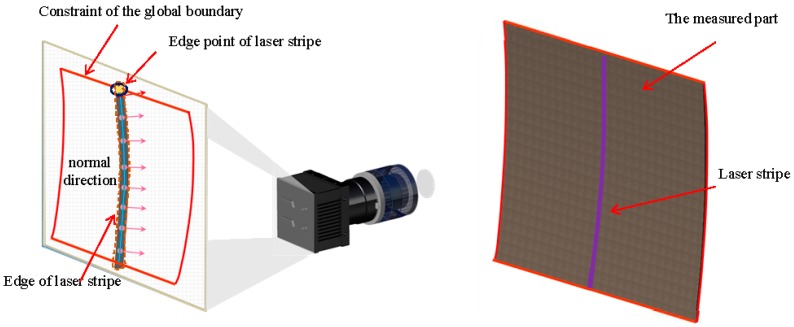
Schematic diagram of laser stripe capture.

Since the laser stripe is the only characteristic information in the measurement process, its extraction accuracy directly determines the surface measurement accuracy of a large part [18]. However, the aviation part is both large and has a rough surface. Further, the illumination in industrial environments is complex. Consequently, capturing high-quality images of laser stripes is difficult, and even the Gaussian characteristic of the laser stripe on the cross section is virtually lost. In addition, because the background of the measurement field is complicated and changeable, the laser stripes projected onto the boundary of the object are not subject to any specific rules. As a result, high-accuracy extraction of the laser stripe on the boundary is difficult to achieve. To overcome this problem, the extraction method of edge points is proposed in this paper which is based on global boundary constraint and the analysis of local stripe feature. First, extraction is carried out based on compression processing to obtain the center of the laser stripes with high accuracy and less amount of calculation. Then, the global boundary of the part is extracted to build the global boundary constraint. Finally, based on the global boundary constraint, the edge points of the laser stripe center are determined. A block diagram of the proposed measuring method and laser stripe capture process is shown in Figure 1. The process of laser-stripe capture is shown in Figure 2.

## 3. Extraction Method

### 3.1. Extraction of Laser Stripe Center

#### 3.1.1. Sub-Pixel Centroid Extraction Based on Compress Processing

In order to meet the aviation parts measurement requirements with high accuracy and speed in the industrial field, the laser stripe center should be extracted highly accurately in the least amount of time possible. Thus, an improved extraction method of laser stripe with high accuracy and less calculation is very important for the achievement of aviation parts’ measurement.

**Figure 3:**
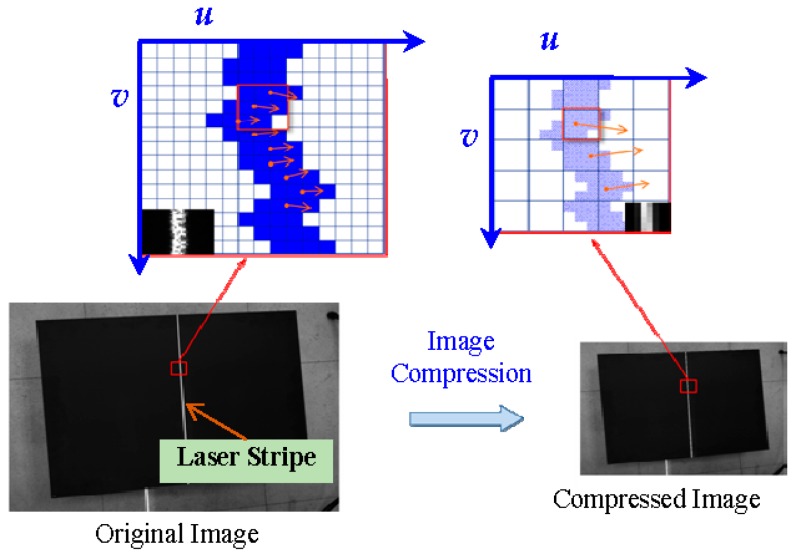
The image compression principle (the compression ratio, λ, in this Figure is 4).

Image compression technology can remove the interference information in the original image and retain the structural feature of the measured part [19]. Based on the compression processing technology, a sub-pixel centroid extraction method based on compress processing is proposed in this paper. The proposed method improves extraction accuracy in the industrial field and reduces the time taken to compute the normal direction. In the proposed method, the normal direction of the stripes is calculated using the low-resolution image compressed from the original image based on image compression technology to minimize the computation time. Combined with the stability of the normal direction and the presented judgment criterion of the normal center, the sub-pixel center of the laser stripe is extracted rapidly and precisely. The principle is illustrated in Figure 3.

According to the compression ratio, λ, images (resolution m×n) captured by the binocular camera are compressed into low-resolution images. The λ×λ pixels of the image are compressed to one pixel by computing the average value of the local grayscale. As the image resolution number is even, compression factor λ is defined as an even number to simplify the calculation. G is the grayscale matrix of the original image. m and n are the row and column value, respectively. After compressing the image with factor λ, the grayscale matrix of the compressed image can be computed as follows:

(1) $$G^{c}=\frac{1}{\lambda^{2}}\left(M\times G\times N\right)$$

where $G^{c}$ is the grayscale matrix of the compressed image. For more clarity, letters with the superscript c in this paper denote compressed image information. M and N are compression matrixes with scale $\frac{m}{\lambda }\times m$ and $n\times \frac{n}{\lambda }$, respectively. The mathematical expressions can be depicted as follows:

(2) $$M=\left[\begin{array}{cccc} \underset{\lambda }{\underset{︸}{1\cdots 1}} & \underset{\lambda }{\underset{︸}{0\cdots 0}} & \underset{\lambda }{\underset{︸}{0\cdots 0}} & \underset{m-3\lambda }{\underset{︸}{0\cdots 0}}\\ \underset{\lambda }{\underset{︸}{0\cdots 0}} & \underset{\lambda }{\underset{︸}{1\cdots 1}} & \underset{\lambda }{\underset{︸}{0\cdots 0}} & \underset{m-3\lambda }{\underset{︸}{0\cdots 0}}\\ \underset{\lambda }{\underset{︸}{0\cdots 0}} & \underset{\lambda }{\underset{︸}{0\cdots 0}} & \underset{\lambda }{\underset{︸}{1\cdots 1}} & \underset{m-3\lambda }{\underset{︸}{0\cdots 0}}\\ \vdots & \vdots & \vdots & \vdots \\ \underset{m-3\lambda }{\underset{︸}{0\cdots 0}} & \underset{\lambda }{\underset{︸}{0\cdots 0}} & \underset{\lambda }{\underset{︸}{0\cdots 0}} & \underset{\lambda }{\underset{︸}{1\cdots 1}} \end{array}\right],N={\left[\begin{array}{cccc} \underset{\lambda }{\underset{︸}{1\cdots 1}} & \underset{\lambda }{\underset{︸}{0\cdots 0}} & \underset{\lambda }{\underset{︸}{0\cdots 0}} & \underset{n-3\lambda }{\underset{︸}{0\cdots 0}}\\ \underset{\lambda }{\underset{︸}{0\cdots 0}} & \underset{\lambda }{\underset{︸}{1\cdots 1}} & \underset{\lambda }{\underset{︸}{0\cdots 0}} & \underset{n-3\lambda }{\underset{︸}{0\cdots 0}}\\ \underset{\lambda }{\underset{︸}{0\cdots 0}} & \underset{\lambda }{\underset{︸}{0\cdots 0}} & \underset{\lambda }{\underset{︸}{1\cdots 1}} & \underset{n-3\lambda }{\underset{︸}{0\cdots 0}}\\ \vdots & \vdots & \vdots & \vdots \\ \underset{n-3\lambda }{\underset{︸}{0\cdots 0}} & \underset{\lambda }{\underset{︸}{0\cdots 0}} & \underset{\lambda }{\underset{︸}{0\cdots 0}} & \underset{\lambda }{\underset{︸}{1\cdots 1}} \end{array}\right]}^{T}$$

The compression image is shown in Figure 3. As the structural features of the laser stripes primarily remain after compressing the original image, the normal vector of the stripe can be computed using the compressed image to reduce the calculation time. First, the stripe centers of the low-resolution image are preliminarily extracted using the gray centroid method. The initial values of the centers are used to compute the normal vector of the laser stripe. Thus, the initial center values of the compressed image are set as $C_{i}^{c}=\left(\begin{array}{cc} V_{i}^{c} & U_{i}^{c} \end{array}\right)$. $V_{i}^{c}$ and $U_{i}^{c}$ are the values of the row and column, respectively. The normal vectors of the laser stripe are calculated by fitting the extracted initial center points. As the curvature variation is small in a small region, the polynomial fitting method is used to fit the curve of initial centers. To calculate the normal vector of the stripe center on the ith row, the center of the ith row and centers of the σ rows before and after the ith row are selected. The number of selected points should be greater than four to ensure fitting accuracy. The set of selected points is as follows:

(3) $$D_{i}^{c}=\left\{C_{w}^{c}|i-\sigma \leq w\leq i+\sigma ,w\in {Ν}^{*},\sigma \geq 3\right\}$$

where ${Ν}^{*}$ is a set of positive integers. Based on the ith-row set of selected points, the fitting equation of the curve of stripe centers can be established as follows:

(4) $$F_{i}^{c}(u,v)=v-\sum_{\omega =0}^{t}a_{i,\omega }u^{\left(t-\omega \right)}=0$$

where $a_{i,\omega }$ is the polynomial fitting coefficient and v,u are the row and column variable, respectively. t is the maximal optional polynomial order. Then, the normal vector $\nabla F_{i}$ satisfies the following equation:

(5) $$\nabla F_{i}^{c}=\{F_{iu}^{c},F_{iv}^{c}\}$$

where $F_{iu}^{c}$ and $F_{iv}^{c}$ are partial derivatives of $F_{i}^{c}(u,v)$ to column u  and row v, respectively. Therefore, $F_{iu}^{c}$ and $F_{iv}^{c}$ can be expressed as follows:

(6) $$F_{iu}^{c}=(t-j)\sum_{j=0}^{t-1}a_{i,\omega }u^{\left(t-j-1\right)},F_{iv}^{c}=1$$

Subsequently, using the hierarchical calculation method, the normal vectors obtained in the compressed image can be restored to the original image. According to the principle of compression, one row of pixels in the compressed image represents λ rows in the original image. Thus, the normal vector at each row of laser stripe in the compressed image is defined as the one at the (λ+1)/2 row of laser stripe in the original image:

(7) $$\nabla F_{i}^{c}=\nabla F_{\lambda (i-1)+\left(\lambda +1\right)/2}$$

$\nabla F_{\lambda (i-1)+\left(\lambda +1\right)/2}$ is the normal vector of the center in the row λ(i−1)+(λ+1)/2 of the original image. According to the normal vector of the rows before and after the selected row in the compressed image, the normal vectors of the middle rows in the original image can be acquired via linear interpolation. The computation equation is as follows:

(8) $$\nabla F_{j}=\{\nabla F_{ju},\nabla F_{jv}\}=\nabla F_{\lambda (i-1)+\lambda /2+h}=\frac{\nabla F_{i}^{c}}{\left|\nabla F_{i}^{c}\right|}+\frac{\nabla F_{i+1}^{c}}{\left|\nabla F_{i+1}^{c}\right|}\times \frac{h-1/2}{\lambda /2},\;h=1,2,3,\ldots ,\lambda$$

therefore, stripe slope $k_{j}$ of the stripe center on the jth row of the original image can be calculated as follows:

(9) $$k_{j}=\nabla F_{jv}/\nabla F_{ju}$$

**Figure 4:**
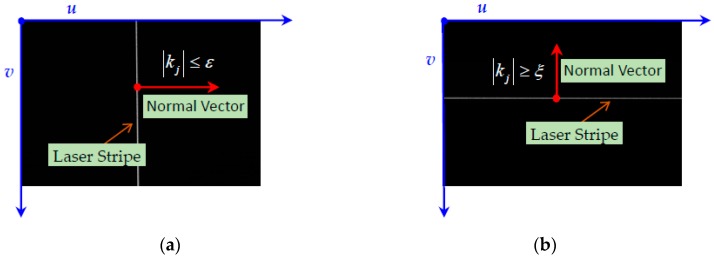
Different normal slope value situations: (**a**) parallel with column; and (**b**) parallel with row.

The stripes slopes have two special limiting values: infinity and infinitely close to zero. When the slope is infinitely close to zero, specifically, $\left|k_{j}\right|\leq \varepsilon (\varepsilon =10^{-10})$, the normal vector of laser stripe is approximately parallel to the column vector of the image. In this case, the grayscale values of the stripe are ascertained along with the column direction of the image. When the slope is close to infinity, namely $\left|k_{j}\right|\geq \xi (\xi =10^{10})$, the normal direction is approximately parallel to the row vector of the image. In this case, the grayscale values of the stripe are ascertained along with the row direction of the image. With the exception of these two special situations, the grayscale values of the stripe are ascertained along with the normal direction. The two special situations are as shown in Figure 4.

**Figure 5:**
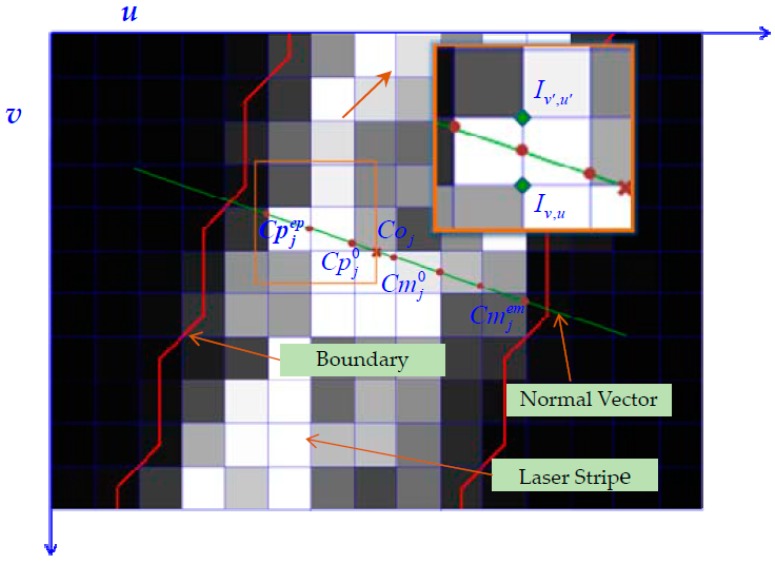
Gray centroid judgment criterion.

After calculating the normal slopes, the centers of the laser stripes on the original image are extracted. First, the centers are preliminarily extracted. In the process of image acquisition, the quality of the images is affected by noise caused by various factors such as the quality of the sensor and ambient light. Therefore, a Gaussian filter is used to preprocess the image before stripe center extraction to remove some of the image noise. Then, the original image is binarized and the edge of the laser stripe is extracted [20]. The sequence of the points on the edge is denoted as B. Next, the centroid of gray method is used to calculate the pixel coordinates of the stripe centers on the original image. To facilitate subsequent analysis and calculation, the pixel coordinates of the centers are rounded off. The rounded values are defined as the initial values of the stripe centers of the original image, denoted as $Co_{j}=\left(\begin{array}{cc} Vo_{j} & Uo_{j} \end{array}\right)$. Combined with the initial values of the stripe centers and their corresponding normal slopes, the grayscale information in the normal direction of the original image is determined, and the gray centroid along the normal direction of the stripe is obtained. Traditionally, the stripe center is acquired by using the algorithm of COG in normal direction at one row of the laser stripe. However, because the quality of the captured image is poor in an industrial setting, the extraction accuracy of the stripe center is easily affected by error points. Consequently, in this paper, interpolation and edge judgment are adopted to improve the judgment criterion of the normal pixels and to precisely calculate the gray centroid. The following procedure is utilized. The process of Gray centroid judgment is shown in Figure 5.

##### Determine the Points on the Normal Direction of the Laser Stripes

The normal line of the stripe intersects with both the row and column of the image. As selecting all these intersections would increase the computation burden, owing to the significant amounts of redundant data present, considering the characteristics of the laser image, the search is conducted along the intersections of the row or column, which results in more intersections without redundant data. The sequence of the stripe slopes $K=\{k_{j}|j=1,2,3,\ldots ,m\}$ is the set of normal slopes of the initial center point in each row of the original image. Then, the numbers of $\left|k_{j}\right|<1$ and $\left|k_{j}\right|>1$ in the sequence K are calculated, denoted as $N_{K<1}$ and $N_{K>1}$ respectively. When $N_{K<1}>N_{K>1}$, the stripe has more normal vectors with a smaller angle in the column direction. The stripe is defined as a column stripe. The length of these laser stripes is distributed in the row and the width is distributed in the column. Therefore, the pixel points for these stripes are sought in the column. Conversely, when $N_{K<1}<N_{K>1}$, the stripes can be defined as row stripes, and so they are sought in the row.

##### Select Pixel Points

Let us take a column stripe as an example stripe that is sought by the column. The pixel-point selection method is as follows: the preliminary stripe centers of the original image $Co_{j}=\left(\begin{array}{cc} Vo_{j} & Uo_{j} \end{array}\right)$ are taken as the initial points. Then, $Uo_{j}$ is rounded to infinity and zero, respectively, denoted as $Up_{j}^{0}$ and $Um_{j}^{0}$. Next, the pixel points are sought along the normal direction where the slope of the normal line is $k_{j}$ according to the manner in which the column increases and decreases. Then, the pixel points where the stripe normal intersects with the column vector of the original image can be determined. Therefore, the column vector in the increasing and decreasing direction can be obtained as $Up_{j}^{s}$ and $Um_{j}^{s}$, respectively:

(10) $$Up_{j}^{s+1}=Up_{j}^{s}+1,Um_{j}^{s+1}=Um_{j}^{s}-1,s=0,1,2,3\ldots$$

The row vector where the stripe normal intersects with the column vector is

(11) $$Vp_{j}^{s}=k_{j}\left(Up_{j}^{s}-Uo_{j}\right)+Vo_{j},Vm_{j}^{s}=k_{j}\left(Um_{j}^{s}-Uo_{j}\right)+Vo_{j}$$

Then, the sets of selected points in the increasing and the decreasing direction are expressed as $Dp_{j}$ and $Dm_{j}$, respectively:

(12) $$\begin{array}{l} Dp_{j}=\left\{Cp_{j}^{s}|Cp_{j}^{s}=\left(\begin{array}{cc} Vp_{j}^{s} & Up_{j}^{s} \end{array}\right),0<Vp_{j}^{s}\leq m,0<Up_{j}^{s}\leq n,s=0,1,2,3\ldots \right\}\\ Dm_{j}=\left\{Cm_{j}^{s}|Cm_{j}^{s}=\left(\begin{array}{cc} Vm_{j}^{s} & Um_{j}^{s} \end{array}\right),0<Vm_{j}^{s}\leq m,0<Um_{j}^{s}\leq n,s=0,1,2,3\ldots \right\} \end{array}$$

where $Cp_{j}^{s}$ and $Cm_{j}^{s}$ are, respectively, the selected pixel center in the increasing and the decreasing direction. Then, two critical points are obtained, which are the intersection points between the edge of laser stripe and the normal line of laser stripe. Since the edge of laser stripe is composed of discrete points, the extraction criterion of critical points is as follows. Firstly, extreme points of stripe edge at each row are recorded by using parameters $U_{h}^{\max}$ and $U_{h}^{\min}$. They are the maximum and minimum column values of stripe edge at the hth row of image, respectively. According to extreme points, the critical points are determined. To facilitate this method introduction, the calculation method of critical point that belongs to the sequence of selected points. $\left\{Cp_{j}^{s}\right\}$ in the increasing direction is taken as an example. $Cp_{j}^{0}=\left(\begin{array}{cc} Vp_{j}^{0} & Up_{j}^{0} \end{array}\right)$ is the initial point. Row vector $Vp_{j}^{0}$ is rounded to $\bar{Vp_{j}^{0}}$ and $\bar{Vp_{j}^{0}}$ is marked as h for brevity. When $U_{h}^{\min}\leq Up_{j}^{0}\leq U_{h}^{\max}$ is satisfied, the selected pixel point is the one on the laser stripe. Then, $Cp_{j}^{1}$ is calculated based on Equations (11) and (12). Next, the edge condition of $Cp_{j}^{1}$ is determined in the same manner as the initial point. The iterative calculation is terminated when $h=\bar{Vp_{j}^{pm+1}}$, $Up_{j}^{pm+1}<U_{h}^{\min}orUp_{j}^{pm+1}>U_{h}^{\max}$. The critical point in the increasing direction is denoted as $Cp_{j}^{ep}$. The one in the decreasing direction $Cm_{j}^{em}$ is obtained in the same manner. The equation is

(13) $$\begin{array}{l} Dp_{j}=\left\{Cp_{j}^{s}|Cp_{j}^{s}=\left(\begin{array}{cc} Vp_{j}^{s} & Up_{j}^{s} \end{array}\right),0<Vp_{j}^{s}\leq m,0<Up_{j}^{s}\leq n,s=0,1,2,3\ldots ep\right\}\\ Dm_{j}=\left\{Cm_{j}^{s}|Cm_{j}^{s}=\left(\begin{array}{cc} Vm_{j}^{s} & Um_{j}^{s} \end{array}\right),0<Vm_{j}^{s}\leq m,0<Um_{j}^{s}\leq n,s=0,1,2,3\ldots em\right\} \end{array}$$

Finally, the set of selected pixel points in the jth row is arranged as $D_{j}=\left\{C_{j}^{s}|C_{j}^{s}=\left(\begin{array}{cc} V_{j}^{s} & U_{j}^{s} \end{array}\right),s=1,2,3,\ldots \right\}$: when $Uo_{j}$ (the column vector of the initial center) is non-integer, we can get $Up_{j}^{0}\neq Um_{j}^{0}$. Thus, $D_{j}=Dm_{j}\cup Dp_{j}$ is chosen as the set of pixel points; when $Uo_{j}$ is an integer, $Up_{j}^{0}=Um_{j}^{0}$ is obtained. The set of selected pixel points without repeat points $Cp_{j}^{0}$ is chosen. This is denoted as $D_{j}=Dm_{j}\cup Dp_{j}-\{Cp_{j}^{0}\}$.

##### Calculate the Grayscale of the Selected Point

Since the selected points are obtained via the sub-pixel algorithm, the grayscales of selected points should be calculated via interpolation. The row value $V_{j}^{s}$ of selected pixel point $C_{j}^{s}$ is rounded towards plus infinity and zero, respectively, which are denoted as $V_{j}^{s}\left(one\right)$ and $V_{j}^{s}\left(zero\right)$. If $V_{j}^{s}\left(one\right)=V_{j}^{s}\left(zero\right)$, the row vector $V_{j}^{s}$ is an integer. Hence, the grayscale of this point is $I_{j}^{s}$, that is, the grayscale of the selected pixel point is:

(14) $$I_{j}^{s}=I_{v,u},v=V_{j}^{s}\left(one\right),u=U_{j}^{s}$$

where v,u represent the row and column values of the original image. $I_{v,u}$ is the grayscale in the vth row and uth column in the original image. If $V_{j}^{s}\left(one\right)\neq V_{j}^{s}\left(zero\right)$, the interpolation method is used to calculate the grayscale:

(15) $$\begin{array}{l} I_{j}^{s}=\left(V_{j}^{s}-V_{j}^{s}\left(zero\right)\right)\times \left(I_{v^{\prime },u^{\prime }}-I_{v,u}\right)+I_{v,u}\\ v=V_{j}^{s}\left(zero\right),v^{\prime }=V_{j}^{s}\left(one\right),u=u^{\prime }=U_{j}^{s} \end{array}$$

where $I_{v^{\prime },u^{\prime }}$ and $I_{v,u}$ are the grayscales in $\left(V_{j}^{s}(\mathrm{one}),U_{j}^{s}\right)$ and $\left(V_{j}^{s}(\mathrm{zero}),U_{j}^{s}\right)$ of the original image, respectively. There is an exceptional situation in which the row vector of the selected point is the extreme value of the image row; that is, $V_{j}^{s}<1$. In this case, the gray value $I_{j}^{s}$ is defined as the value where $V_{j}^{s}=1$.

##### Extract the Gray Centroid

On the basis of the above calculation, the gray centroid in the normal direction of the initial point in the jth row of the original image has been calculated. In addition, the corresponding grayscale $I_{j}^{s}$ of the selected pixel points $C_{j}^{s}$ have been obtained. Thus, the gray centroid $C_{j}$ in the row can be extracted based on the results of the selected pixel points and corresponding grayscale. The equation is as follows:

(16) $$C_{j}=\sum_{s=1}^{\eta }\left(C_{j}^{s}I_{j}^{s}\right)/\left(\sum_{s=1}^{\eta }I_{j}^{s}\right)$$

where η is the number of the selected pixel points $C_{j}^{s}$.

#### 3.1.2. Edge-Point Extraction Based on Directed Arc-Length Criterion

In the manufacturing process, the boundary of the aerodynamic shape is usually used as the geodetic datum owing to the deformation of a large aviation space. Thus, accurate extraction of the boundary is critical to the detection of aviation parts. Because the measuring environments in the industrial field are complicated, much noise is generated in the process of image acquisition. Thus, the quality of the images captured in industrial environments is poor, especially in the boundary region. Conventionally, the noise is reduced using data analysis software. However, some information of boundary of the measured object is lost as a result of deleting the points with a certain distance from the measuring plane. In order to reconstruct the aerodynamic shape more accurately, the boundary of the parts should be precisely extracted to retain the geometric features of the parts more completely. Therefore, a method based on global boundary constraint and the analysis of local stripe feature is proposed in this paper to obtain more accurate information about the laser stripes on the edge of the parts.

Carbon fiber composite material is widely used in the manufacture of aviation parts. Because the surface of the composite material is black, absorbance is high. When the light entering the camera is increased, the camera gets more of the light reflected from the background than the light reflected from the surface of the part. Thus, there is a substantial contrast between the part and background. Using this high contrast, a global boundary is first extracted in this study as constraints of the edges of the laser stripes.

The global boundary image acquisition method is as follows. Before scanning the laser stripes, the exposure time of the camera is increased. With added light into the camera, a non-stripe image with a large contrast between the boundary of the part and the background is captured based on the surface characteristic of the carbon fiber. If the reflectance of the background is low, reflective tapes can be pasted on the border area of the parts’ back. Therefore, the boundary of the part has a high contrast with the background of the image. The boundary constraint of the laser stripe can be established by extracting the entire boundary of the part. The procedure is as follows: First, the region of the part is selected to establish the region of interest and reduce the quantum of calculation. Then, the image is processed using a median filter to denoise it. Following binarization of the image and deletion of the small/big area affected by noise, the global boundaries of the part are preliminarily extracted using an edge-detector algorithm. Finally, the boundaries of the parts are confirmed via the intersection seek method and marked as $B_{g}=\{Q_{\alpha }=(u_{\alpha },v_{\alpha })|\alpha =1,2,3,\ldots \}$, where $Q_{\alpha }(u,v)$ is the pixel coordinates of the extracted points of the boundary.

**Figure 6:**
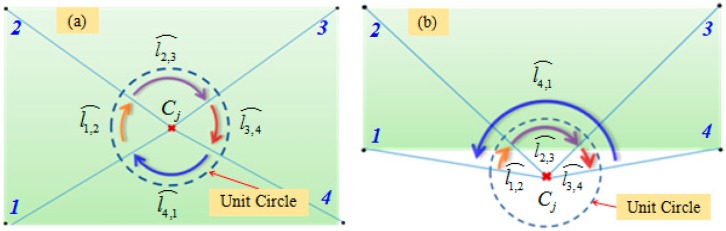
Schematic diagram of the directed arc-length method: (**a**) point inside the boundary; and (**b**) point outside the boundary.

Based on the constraint of the global boundary, a point on the local edge is determined from the extracted centers of scanning laser stripes. In this paper, an edge-point extraction method based on directed arc-length criterion is adopted to determine whether the laser stripe centers are within the global boundary. The principle of the arc-length method is to calculate the sum of the directed arc-lengths that the edge points projected onto a unit circle with the center of the judged point. The principle is shown in Figure 6. The graph enclosed by the boundary is closed and directed. A unit circle is constituted with the center of the judged point. The edge points are connected to the center in accordance with a certain sequence. The intersections of these connections and the unit circle are defined as projection points. In accordance with the sequence, the directed unit arc-lengths of the projection points are summed in a manner that the angle is less than or equal to 180 degrees. If the judged point is on or within the boundary, the sum of the directed arc length is 2π, and the point is called an interior point; if it is outside of the global boundary, the sum is zero.

Because of the large amount of laser stripe centers and boundary constraint, we improved the method to determine the point of the local edge based on the arc-length method. For convenient introduction, the column laser stripe is taken as an example. The extracted center of the laser stripe is arranged in an extraction order with the increasing of the column value of the image. Therefore, the points of the edge are distributed on the regions of the starting and ending, respectively. We judge the extracted light strip centers from the two ends of the center sequence. According to Equation (16), the center sequence of a laser stripe is defined as $\left\{C_{j}|j=1,2,\ldots ,\eta \right\}$. First, the initial point of the center sequence is judged as to whether it is within the boundary, that is, when j=1, $C_{j}$ is judged whether it has met the requirements of the interior point. Because the boundary consists of thousands of points, the minimum bounding rectangle $REC_{\min}$ of the global boundary is taken as an initial boundary to simplify the calculation. The rectangle is described as follows:

(17) $$REC_{\min}=\left\{\left(\min(v_{\alpha }),\min(u_{\alpha })\right),\left(\min(v_{\alpha }),\max(u_{\alpha })\right),\left(\max(v_{\alpha }),\max(u_{\alpha })\right),\left(\max(v_{\alpha }),\min(u_{\alpha })\right)\right\}$$

Four vertexes are marked as $RE_{1},RE_{2},RE_{3},RE_{4}$, respectively. The sum of the directed arc-length $\overset{⌢}{l_{RE}}$ is calculated as $\overset{⌢}{l_{RE}}=\sum_{i=1}^{3}\overset{⌢}{l_{i,i+1}}+\overset{⌢}{l_{4,1}}$. If the arc-length is zero, $C_{j}$ is not an interior point, and $C_{j+1}$ is judged. If the arc-length is 2π, the directed arc-length of points on the global boundary is summed:

(18) $$\overset{⌢}{l_{g}}=\sum_{i=1}^{\eta -1}\overset{⌢}{l_{i,i+1}}+\overset{⌢}{l_{\eta ,1}}$$

if $\overset{⌢}{l_{g}}$ does not meet the requirements of the interior point, the next point is sequentially judged. The calculation continues until the inner point appears. The inner point is considered as a boundary point $C_{f}$ of the laser stripe. Similarly, another edge point is judged from another end of the center sequence. Then, a series of laser stripe centers $\left\{C_{j}|j=f,f+1,\ldots ,l-1,l\right\}$ is reserved as the center points within the boundary. Parameters f and l are the first number and last number, respectively.

### 3.2. Feature Reconstruction

#### 3.2.1. System Calibration

High-accuracy vision measurement system calibration is the key and foundation to ensure the measurement accuracy of large parts. In this paper, the binocular system is calibrated using the two-dimensional target calibration method presented by Zhang [21]. In order to reduce the influence of the lens distortion on measurement accuracy, the correction coefficient for the radial distortion is introduced. Further, the pinhole model is modified to ensure high-accuracy calibration of the high-speed camera [7]. The corresponding equation is as follows:

(19) $$Z_{c}\left[\begin{array}{l} \hat{u}\\ \hat{v}\\ 1 \end{array}\right]+Z_{c}\left[\begin{array}{ccc} k_{1}\hat{u} & k_{2}\hat{u} & 0\\ k_{1}\hat{v} & k_{2}\hat{v} & 0\\ 0 & 0 & 1 \end{array}\right]\left[\begin{array}{c} {\hat{u}}^{2}+{\hat{v}}^{2}\\ {\left({\hat{u}}^{2}+{\hat{v}}^{2}\right)}^{2}\\ 1 \end{array}\right]=M^{l}\left[\begin{array}{l} X_{w}\\ Y_{w}\\ Z_{w}\\ 1 \end{array}\right]$$

where, $\left(\begin{array}{cc} \hat{u} & \hat{v} \end{array}\right)$ is the real image point caused by distortion, and $k_{1},k_{2}$ are known as the first-and second-order distortion coefficients. $\left(\begin{array}{ccc} X_{w} & Y_{w} & Z_{w} \end{array}\right)$ is the three-dimensional coordinates of an image point in the world coordinate system; $Z_{c}$ is a scale factor; $M^{l}$ represents the camera parameters. Calibration of the binocular camera from two dimensions to three dimensions is achieved via LM optimization.

#### 3.2.2. Features Matching and Reconstruction

**Figure 7:**
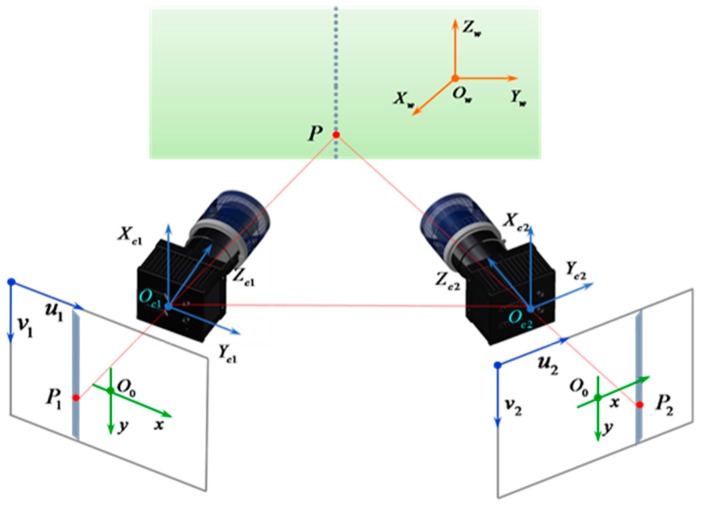
Illustration of the matching and reconstruction process.

The matching and reconstruction of corresponding feature points on the left and right images are essential to realizing high-accuracy 3D measurements. The illustration of the matching and reconstruction process is shown in Figure 7.

Based on basic epipolar constraint, the corresponding points of laser stripe centers on the left image and right image are matched [22]. The parameters of matching are obtained by using the 8-point algorithm [23]. Then the matching points are reconstructed according to the basic principle of binocular vision reconstruction and the parameters of reconstruction are obtained based on the results of camera calibration [24]. The 3Dcoordinates of a point $P_{i}$ are reconstructed based on the following equation:

(20) $$\begin{array}{l} X_{w}m_{11}^{l}+Y_{w}m_{12}^{l}+Z_{w}m_{13}^{l}+m_{14}^{l}-u^{l}X_{w}m_{31}^{l}-u^{l}Y_{w}m_{32}^{l}-u^{l}Z_{w}m_{33}^{l}=u^{l}m_{34}^{l}\\ X_{w}m_{21}^{l}+Y_{w}m_{22}^{l}+Z_{w}m_{23}^{l}+m_{24}^{l}-v^{l}X_{w}m_{31}^{l}-v^{l}Y_{w}m_{32}^{l}-v^{l}Z_{w}m_{33}^{l}=v^{l}m_{34}^{l}\\ X_{w}m_{11}^{r}+Y_{w}m_{12}^{r}+Z_{w}m_{13}^{r}+m_{14}^{r}-u^{r}X_{w}m_{31}^{r}-u^{r}Y_{w}m_{32}^{r}-u^{r}Z_{w}m_{33}^{r}=u^{r}m_{34}^{r}\\ X_{w}m_{21}^{r}+Y_{w}m_{22}^{r}+Z_{w}m_{23}^{r}+m_{24}^{r}-v^{r}X_{w}m_{31}^{r}-v^{r}Y_{w}m_{32}^{r}-v^{r}Z_{w}m_{33}^{r}=v^{r}m_{34}^{r} \end{array}$$

where $m_{ij}^{l}$ is the value on the ith row and jth column of matrix $M^{l}$, and $M^{l}$ is the projection matrix of the left camera, which expresses the relationship between 2D and 3D on the left camera; $u^{l},v^{l}$ are image pixel coordinates of left camera image on the column and row, respectively; $m_{ij}^{r}$ is the value of the right camera projection matrix $M^{r}$ on the ith row and jth column; $u^{r},v^{r}$ are image pixel coordinates of the right camera; and the pixel points of the left and right image are the matching points. $X_{w},Y_{w},Z_{w}$ are the final 3D coordinates of the measured point. Based on above proposed method, the 3D surfaces of the measured parts are finally obtained by reconstructing image sequences of the laser stripes captured by the left and right cameras.

## 4. Experimental Analysis

**Figure 8:**
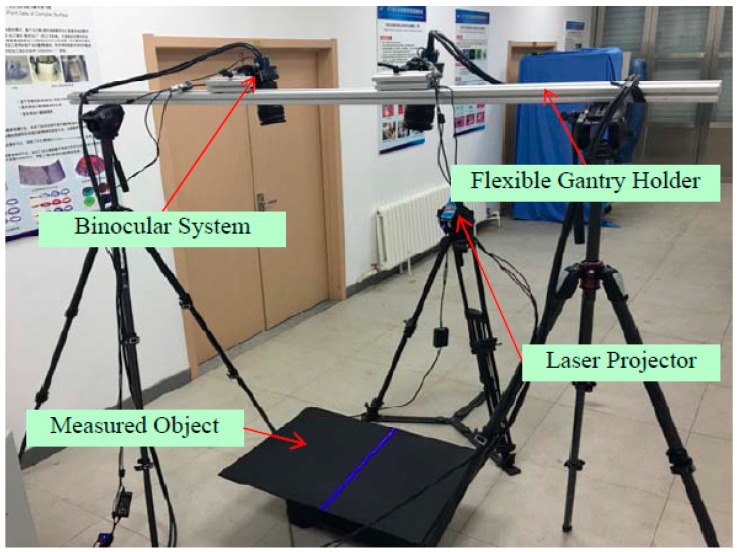
The experimental system used for high-precision measurement.

The experimental system utilized is shown in Figure 8. The binocular vision measurement system for large parts consisted of two high-resolution cameras (VC-12MC with resolution 4096 × 3072 and 35 mm lenses, Vieworks, Gyeonggi-do, Korean), a laser line projector (Lasiris PowerLine with wavelength 445 nm ± 10 nm, Coherent, Salem, NH, USA), gantryholder (Manfrotto tripods MT055CXPRO3 with working height of 1.7 m), and an imaging workstation (Z840, Hewlett-Packard Development Company, Palo Alto, CA, USA). The camera had a field of view of 1270 mm × 700 mm. The baseline of two cameras is 750 mm and the angel of camera is about 30 degree in the proposed system. The configuration of the system is based on the layout optimization algorithm [25]. The measured object was placed parallel to the ground, similar to the placement of aviation parts in the industrial field.

### 4.1. Physical Experiments

**Figure 9:**
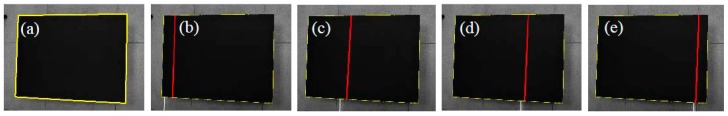
Images captured by the left camera: (**a**) the first image without laser stripe; (**b**) the 10th image; (**c**) the 30th image; (**d**) the 50th image; (**e**) the 70th image.

**Figure 10:**
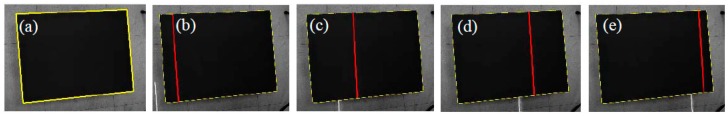
Images captured by the right camera: Images captured by the right camera: (**a**) the first image without laser stripe; (**b**) the 10th image; (**c**) the 30th image; (**d**) the 50th image; (**e**) the 70th image.

A standard flat part with size 600 mm × 800 mm was measured to verify the 3D measurement accuracy of the proposed system. Before the experiments, the standard flat part was measured using a three-coordinate measuring machine (Zeiss Prismo Navigator) to obtain the measuring reference data. Subsequently, the standard flat part was measured using the proposed system. The following experimental procedure was employed. First, the global boundary of the measured parts was obtained from the captured images without laser stripes. The extracted boundaries in the images, captured using left and right cameras, are shown in Figure 9a and Figure 10a. Next, the images of the scanning laser stripes were captured via binocular camera with a frame rate of 20 fps in a time of 4 s. Then, the centers of the laser stripes were extracted by using the extraction method proposed in this paper. In order to compare the measurement accuracy, the centers of the laser stripes were also extracted using common methods—COG method and geometric center method—which are widely used in industrial measurement. Finally, the mean distance deviation (MMD) of the flat surface was utilized to evaluate the measurement accuracy due to the flat characteristic of the standard part.

An original image and four arbitrary captured images, out of a total of 80, are shown in Figure 9. Figure 9a shows the first images captured by the left camera with a longer exposure time and no laser stripe. Figure 9b–e shows the 10th, 30th, 50th and 70th images captured by the left camera, respectively. The corresponding images captured by right camera are shown in Figure 10a–e. The extracted centers of the laser stripes and global boundary based on the proposed method are also indicated in Figure 9 and Figure 10. The red lines represent the extracted center, whereas the yellow curve signifies the global boundary.

**Figure 11:**
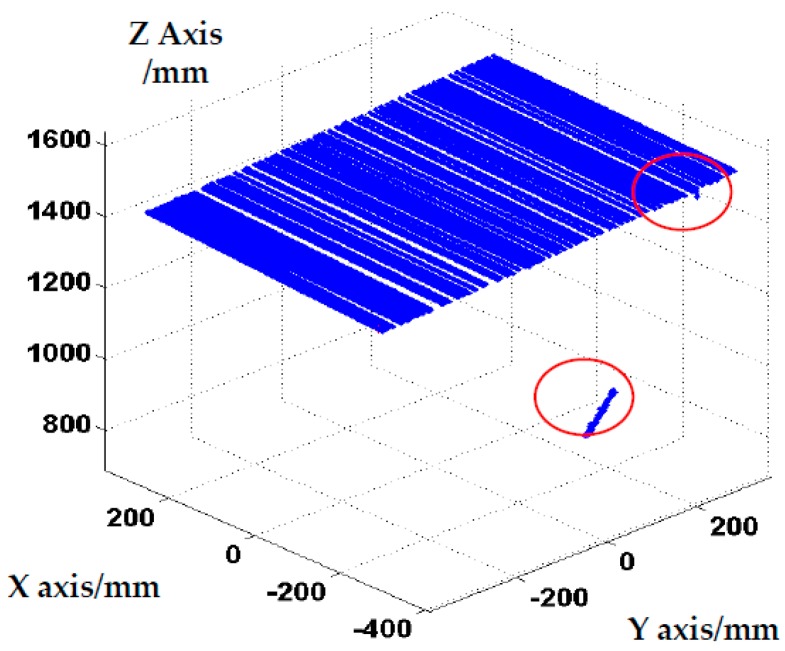
Reconstruction results without global boundary constraint.

**Figure 12:**
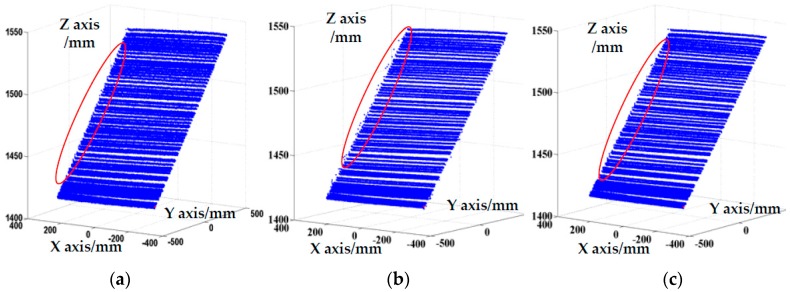
Reconstruction results with different extraction methods. (**a**) Geometric center method; (**b**) Centroid of Gray (COG); (**c**) feature compression extraction method.

**Figure 13:**
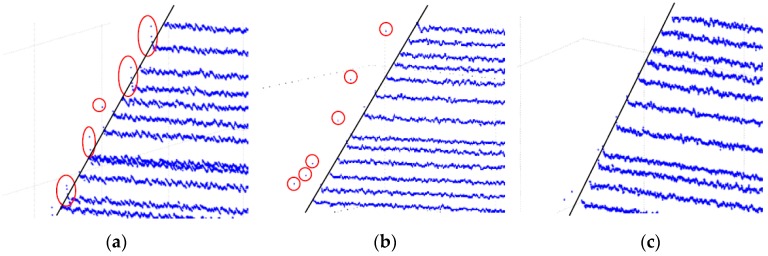
Local reconstruction details on the boundary. (**a**) Geometric center method; (**b**) Centroid of Gray (COG); (**c**) feature compression extraction method.

The scanning laser stripes without the global boundary constraint produced the reconstruction results shown in Figure 11. As shown in the Figure 11, some obvious error points exist on the boundary of object and false stripes present in the reconstruction results (marked with a red circle). The results validate that the proposed method with global boundary constraint can effectively improve the accuracy of reconstruction. The results of the 3D reconstruction based on the method of COG, the method of geometric center, and the feature compression extraction method (proposed in this paper) are given in Figure 12a–c. The local reconstruction details in the boundary are shown in Figure 13. As shown in Figure 12a, there appear many mistake points in the object boundary with the extraction of geometric center method. The boundary is not smooth and the reconstruction boundary points greatly fluctuate. Since the extraction of one-dimensional sub-pixel centers is achieved by the method of COG, the reconstruction errors in the object boundary obviously are reduced. However a portion of gross error points appear on the boundary of object, which is shown in Figure 12b and Figure 13b. In contrast to these two methods, the boundary extracted by the improved center extraction method proposed in this paper is a flat line with fewer mistake points and the deviation of mistake points is smaller.

In order to intuitively compare the reconstruction precision of the three methods, an evaluation method called mean distance deviation (MDD) is proposed. Because the experiment standard part is a flat part, the ideal reconstruction result is a standard plane. However, actual reconstruction points deviate from the plan due to the inevitable reconstruction errors. Therefore, in this paper, mean distance deviation (MDD) of 3D reconstruction point cloud to the fitting plane is defined to evaluate the reconstruction accuracy. The equation of a plane can be fitted by the least square method as Ax+By+Cz+D=0, and the reconstructed points are presented as $p_{i}=\left(\begin{array}{ccc} x_{i} & y_{i} & z_{i} \end{array}\right)$. Thus, the MDD is calculated as follows:

(21) $$\left(\sum_{i=1}^{M}\frac{\left|Ax_{i}+By_{i}+Cz_{i}+D\right|}{{\left(A^{2}+B^{2}+C^{2}\right)}^{1/2}}\right)/M,i=1,2,3,\ldots ,M$$

where M is the number of the reconstructed points. The results are given in Table 1.

The MDD of 4556 points obtained by the three-coordinate measuring machine is 0.10 mm, which is defined as the real MDD. As shown in Table 1, the MDD of the (c) feature compression extraction method proposed in this paper is 0.47 mm. It is smaller than the MDD of the reconstruction results based on the other two methods. The max error is the maximum distance from the reconstructed points to the fitting plane. The maximum error based on the feature compression extraction method is the smallest.

### 4.2. Field Measurement Experiments

The measurement accuracy of the system has been validated through the standard parts in laboratory, while the field measurement of actual parts is conducted to verify the feasibility of the method. In the study of aviation composite parts assembly, loads should be applied at different positions of the part surface. Then combining deformation of the parts with the characteristics of part material and structure, reasonable and effective loading magnitude and positions can be analyzed. Therefore, under different static stress, the shape measurement of the part is important for the analysis of the assembly performance of the composite parts. In order to verify the application feasibility of this method in field measurement, the shape features of a glass fiber composite part surface under different loads are measured by using this method.

**Figure 14:**
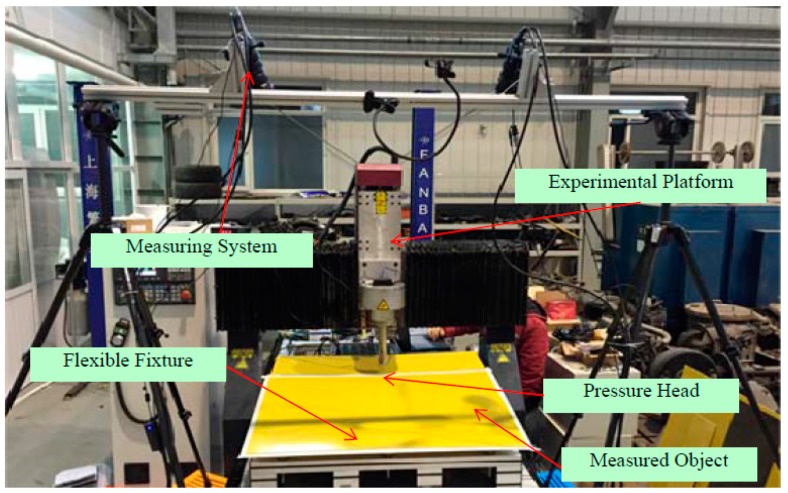
Experimental field measurement setup.

**Figure 15:**
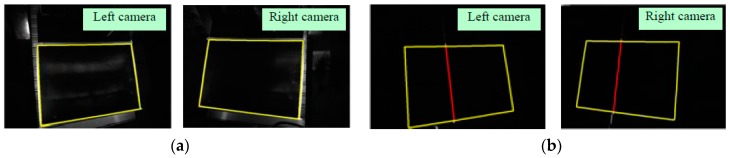
Images captured by the left and right cameras. (**a**) Global boundary obtained with a long exposure; (**b**) the images of laser stripes.

**Figure 16:**
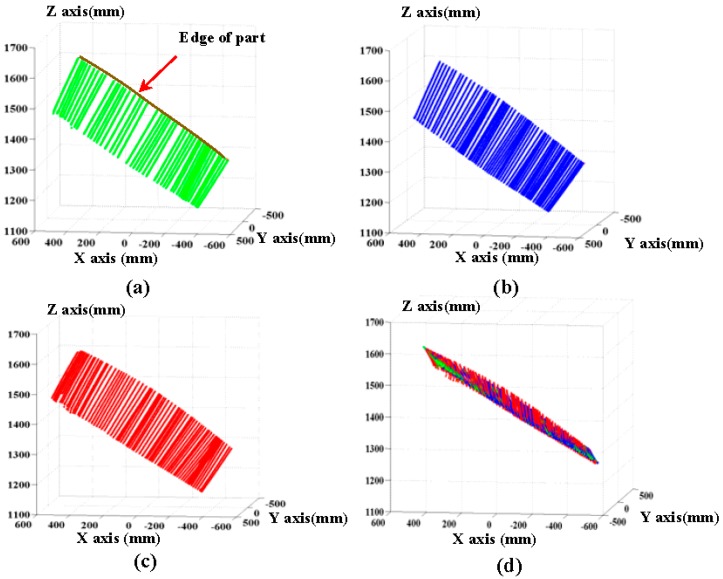
The reconstruction results under different loads: (**a**) the load of 0 N; (**b**) the load of 30 N; (**c**) the load of 50 N; (**d**) the loads of 0 N, 30 N and 50 N.

The size of the aviation part was approximately 700 mm × 800 mm. The field experimental system is shown in Figure 14. When loads are applied onto the surface of an aviation composite part, its 3D shapes change. Based on the proposed system, the three-dimensional shapes of the aviation composite parts under loads of 0 N, 30 N and 50 N are reconstructed respectively. In the field measurement, in order to reduce the impact of the ambient illumination, the lens of the camera is equipped with optical filter. Images captured by the left and right cameras are shown in the Figure 15. The global boundary is obtained with a long exposure. The reconstruction results under different loads are given in Figure 16. The green, blue and red point-clouds are respectively reconstruction results of part under loads of 0 N, 30 N and 50 N, which are shown in Figure 16a–c. The reconstruction results with three loads are shown in Figure 16d.

**Figure 17:**
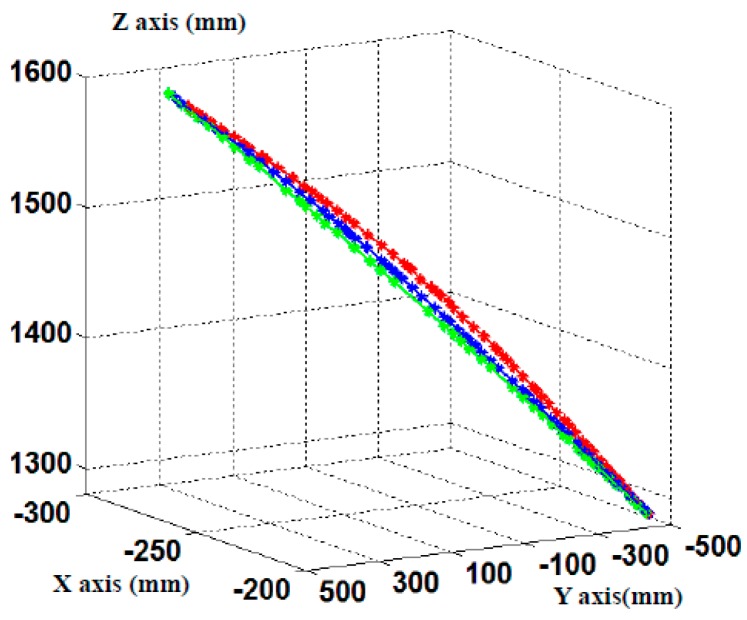
The variation of the measured part’s edge.

The reconstruction results show that the deformation of the part surface increases with the increasing loads. The measurement results conform to the basic deformation law. The variation of the measured part’s edge is shown in Figure 17. The green, blue and red curves are edges of part under loads of 0 N, 30 N and 50 N, respectively. As shown in Figure 17, when 30 N is applied on the one side of the part, a smaller displacement is produced on the middle position of the part’s edge. And when the stress is 50 N, it appears an obvious displacement at the middle position of the part’s edge. However, the two sides of the edge have a small displacement under different loads. The reconstructed point cloud and the edge of part can be used for the analysis of part characteristics with different assembly process parameters.

## 5. Conclusions

In this paper, a large-part measurement method combining with feature compression extraction and directed edge-points criterion is proposed. The proposed method is mainly used to measure surfaces of large aviation parts including boundaries of parts in industrial environments. A sub-pixel centroid extraction method based on compress processing enables the 3D morphology of the measured parts to be accurately reconstructed. In addition, combined with an edge-point extraction based on directed arc-length criterion, the boundary of part is accurately obtained.

The proposed sub-pixel centroid extraction method and directed edge-point criterion improve the extraction accuracy of the laser stripe center and realize the reconstruction of the part’s boundary. Compared with the conventional extraction method, the mean distance deviation of the proposed method is 0.47 mm, which is closest to the real mean distance deviation measured using a coordinate measuring machine. Meanwhile, based on the proposed method, the error points greatly reduced in the region of part’s boundary. Since the measurement of large aviation composite parts has a requirement of non-contact, on-site, less pasted targets and boundary feature in the research process of parts assembly, the proposed method is especially suitable for this measurement in the field. Real measurement experiments of the 3D shapes of aviation composite part under different loads is measured in the field environment validate the effectiveness of the proposed method. However, the measurement accuracy is hard to verify in the field environment due to the lack of suitable verification method of accuracy. Therefore, the analysis method of field measurement accuracy will become an important research direction in the future. Meanwhile, the hardware synchronization and integration of the measurement system will be further improved.

## Figures and Tables

**Table 1:** MDD of Reconstruction.

	Geometric Center Method	Gray Centroid Method	Feature Compression Extraction Method
MDD (mm)	0.60	0.53	0.47
Max Error (mm)	1.13	0.97	0.87
